# Impact of Diabetes Mellitus on Disease Severity and Mortality in Acute Pancreatitis: A Retrospective Single-Center Cohort Study

**DOI:** 10.3390/jcm15020505

**Published:** 2026-01-08

**Authors:** Bayram İnan, Ahmet Akbay, Beril Turan Erdoğan, Çağdaş Erdoğan, İhsan Ateş, Osman Ersoy

**Affiliations:** 1 Department of Gastroenterology, Ankara Bilkent City Hospital, Ankara 06800, Türkiye; oersoy@yahoo.com.tr; 2 Department of Gastroenterology, Gaziantep City Hospital, Gaziantep 27470, Türkiye; dr.ahmetakby@gmail.com; 3 Department of Endocrinology and Metabolism, Ankara City Hospital, Ankara 06800, Türkiye; berituran@hotmail.com; 4 Department of Gastroenterology, School of Medicine, Istinye University, Istanbul 34093, Türkiye; cagdas_erdogan@hotmail.com; 5 Department of Internal Medicine, Ankara Bilkent City Hospital, Ankara 06800, Türkiye; dr.ihsanates@hotmail.com; 6 Department of Gastroenterology, Faculty of Medicine, Ankara Yildirim Beyazit University, Ankara 06800, Türkiye

**Keywords:** diabetes mellitus, acute pancreatitis, disease severity, systemic complications, mortality

## Abstract

**Background:** Diabetes mellitus (DM) is a condition that may increase the severity of acute pancreatitis (AP) through chronic inflammation and disturbances in immune responses. However, the independent effect of DM on clinical outcomes in AP has not yet been fully elucidated. **Methods:** In this retrospective cohort study, 492 patients diagnosed with acute pancreatitis at the Gastroenterology Clinic of Ankara Bilkent City Hospital between January 2022 and March 2025 were included. Patients were divided into two groups based on the presence of diabetes, and outcomes were compared using statistical methods. **Results:** Of the total 492 patients (mean age 58.6 ± 17.2 years; 50.2% female) included, 98 (19.9%) had DM. Moderate-to-severe AP occurred in 67.3% of diabetic versus 37.8% of non-diabetic patients (*p* < 0.0001), and severe disease developed more frequently in the diabetic group (6.1% vs. 1.0%, *p* = 0.0057). Systemic complications were significantly more common in patients with diabetes (45.9% vs. 26.9%, *p* = 0.0004). Hospital mortality was higher among patients with diabetes (9.2% vs. 4.6%, *p* = 0.0344), and Kaplan–Meier analysis demonstrated numerically lower overall survival in patients with diabetes (log-rank *p* = 0.095), with early divergence in survival curves. Cox proportional hazards analysis confirmed diabetes as an independent predictor of in-hospital mortality (adjusted HR 2.64, 95% CI 1.17–5.97; *p* = 0.019). After adjustment for confounders, diabetes remained independently associated with the development of moderate/severe pancreatitis (adjusted OR 2.00, 95% CI 1.24–3.22; *p* = 0.004). Diabetes also independently predicted in-hospital mortality (adjusted OR 3.36, 95% CI 1.35–8.34; *p* = 0.009), along with APACHE II score. ROC analysis demonstrated that adding diabetes mellitus to the APACHE II score significantly improved mortality prediction compared with APACHE II alone (AUC 0.785 vs. 0.724). The retrospective and single-center design of this study may limit its generalizability and create potential selection bias. There were insufficient data on the type of diabetes, its duration, and glycemic control (e.g., HbA1c), and therefore, we could not assess these factors, all of which may influence risk estimates. Although the survival curves showed early divergence, the borderline log-rank significance (*p* = 0.095) highlights the limited statistical power to detect long-term survival differences in this cohort. **Conclusions:** DM is associated with substantially increased severity and in-hospital mortality in AP, primarily through an elevated risk of systemic organ failure. Incorporation of diabetes status into early severity stratification may improve prognostic accuracy and guide closer monitoring and timely interventions in this high-risk population.

## 1. Introduction

Acute pancreatitis (AP) is characterized by a sudden inflammatory process in the pancreas and presents with a clinical picture ranging from mild symptoms to life-threatening multiple organ failure. This disease is known to cause both high morbidity and significant economic burden worldwide due to healthcare expenditures. Key factors affecting the clinical outcomes of AP include the patient’s age, comorbidities, and the severity of the inflammatory response [1,2]. In this context, diabetes mellitus (DM), which belongs to the group of systemic metabolic diseases, is considered an important comorbidity that can affect the course of AP. It has been suggested that immune system disorders, microvascular damage, increased oxidative stress levels, and chronic proinflammatory conditions caused by DM contribute to clinical deterioration in acute inflammatory diseases [3,4]. Studies examining the effect of diabetes mellitus on acute pancreatitis increasingly highlight the role of factors that may increase the severity of the disease, such as duration of diabetes and level of glycemic control. It is thought that long-term diabetes and poor glycemic control may worsen the course of acute inflammatory diseases due to their contribution to chronic inflammation, microvascular damage, and impaired immunity [5,6]. Acute pancreatitis is a disease that can present with different clinical courses depending on the underlying cause, and this can directly affect the prognosis. The most common causes include biliary and alcoholic factors. Diabetes mellitus is thought to be an additional factor that can shape the course of the disease on these different etiological grounds [7]. In particular, it has been suggested that the metabolic and inflammatory changes accompanying diabetes can affect the clinical course and outcomes of acute pancreatitis in different ways depending on the etiology. Diabetes mellitus is often seen in conjunction with obesity, hypertension, dyslipidemia, and cardiovascular diseases, which can independently affect inflammatory responses and clinical outcomes. This cardiometabolic burden can increase systemic stress in acute inflammatory diseases such as acute pancreatitis, thereby exacerbating disease severity. Therefore, the effect of diabetes on acute pancreatitis should be evaluated not in isolation but in conjunction with accompanying comorbidities [8,9]. Although various studies have been conducted on the effects of DM on AP in the current literature, significant differences in findings are noteworthy. This can be explained by limited sample sizes, heterogeneity in patient populations, and methodological diversity in study designs [10]. However, some recently published robust epidemiological data suggest that AP may be more severe in individuals with a diagnosis of DM and that systemic complications develop more frequently in these patients [11,12]. Previous studies have reported conflicting results regarding the impact of DM on the clinical course of AP. However, many of these studies were limited by small sample sizes, insufficient stratification of disease severity, and a lack of survival or prognostic analyses. In the present study, the effects of concomitant DM on disease severity, systemic complication development, length of hospital stay, and mortality were comprehensively evaluated in a large cohort of patients diagnosed with AP. Furthermore, the independent role of diabetes in these clinical outcomes was examined using multivariable statistical analyses, and the hypothesis that DM is a key determinant of poor prognosis in AP was tested.

## 2. Materials and Methods

### 2.1. Patients

This study was designed retrospectively at the gastroenterology department of Ankara Bilkent City Hospital. Hospital records of patients who applied to our hospital between January 2022 and March 2025 were reviewed, and patients diagnosed with AP were registered. Patients who met the AP diagnosis according to the revised Atlanta criteria [13] and were aged 18 years and older were included in this study. The exclusion criteria for these patients were determined as follows: 1—patients with chronic pancreatitis; 2—patients with incomplete clinical and laboratory data; 3—pregnant women. After applying the inclusion and exclusion criteria, a total of 492 patients were included in this study. Diabetes mellitus was considered present if a diagnosis of DM was documented in the patient’s medical records at the time of admission; information regarding diabetes duration, type, or glycemic control was not available and therefore not included in the analysis. The retrospective and single-center design of this study may limit causal inference and the generalizability of the findings. Diabetes-specific details, including diabetes duration, type of treatment, and glycemic control level (e.g., HbA1c), were not included in the analyses because they were not adequately and uniformly recorded in patient records. Furthermore, due to statistical separation (perfect separation), mediation analysis could not be fully performed; this limits the scope of mechanistic inferences. Due to the retrospective design, a priori power analysis could not be performed. Survival follow-up was limited to the in-hospital period. This study was conducted in accordance with the Declaration of Helsinki and was approved by the Ankara Bilkent City Hospital Ethics Committee (TABED 1-25-1696).

### 2.2. Data Collection

All data were obtained retrospectively from the hospital record system. Demographic data, such as age and gender, were recorded for each patient. AP etiologies were recorded as biliary, post-ERCP, alcohol, hypertriglyceridemia, and idiopathic. AP severity was determined as mild, moderate, or severe according to the revised Atlanta criteria. For comorbidities, each patient’s hypertension, DM, and coronary artery disease were recorded. APACHE II, Ranson, BISAP, and HAPS scores were calculated for pancreatitis severity [14,15,16]. Laboratory findings included hemoglobin, white blood cell (WBC) count, platelet count, amylase, lipase, albumin, C-reactive protein (CRP), and creatinine. Hospital length of stay and mortality were recorded for clinical outcomes. Local and systemic complications were recorded for each patient. Local complications were recorded as acute peripancreatic fluid collection, pseudocyst, acute necrotic collection, walled-off necrosis (WON), and pancreatic abscess. Systemic complications were recorded as renal, pulmonary, cardiovascular, gastrointestinal, infection, hematological, neurological, and hepatic systems, along with specific disease types.

### 2.3. Definition

Revised Atlanta criteria: 1—typical abdominal pain (sudden onset, persistent, usual-ly felt in the epigastric region and often radiating to the back, moderate-to-severe ab-dominal pain); 2—elevated serum amylase and/or lipase levels (must be at least three times the upper limit of normal); 3—imaging findings (detection of imaging findings spe-cific to AP using contrast-enhanced computed tomography (CT), magnetic resonance im-aging (MRI), or ultrasonography). If at least two of these three criteria are present, a diagnosis of AP is made [13].

### 2.4. Evaluation of Results

The results were statistically compared by dividing the patients into two groups: AP patients with DM and AP patients without DM.

### 2.5. Statistical Analysis

Continuous variables were presented as the mean ± standard deviation or the median (interquartile range), and categorical variables as counts and percentages. Group comparisons between patients with and without DM were performed using the chi-square or Fisher’s exact test for categorical variables and Student’s *t*-test or Mann–Whitney U test for continuous variables, as appropriate. The primary outcomes were moderate-to-severe AP, defined with the Revised Atlanta Classification, and in-hospital mortality.

Multivariable logistic regression models were constructed to identify independent predictors of these outcomes. Adjusted odds ratios (aORs) with 95% confidence intervals (CIs) were reported. Effect modification for the association between DM and outcomes was evaluated through interaction terms across prespecified subgroups (age < 60 vs. ≥60 years, sex, biliary versus non-biliary etiology, and APACHE II score ≤ 8 vs. >8). In-hospital survival was analyzed using the Kaplan–Meier method and compared using the log-rank test.

To assess prognostic performance, receiver operating characteristic (ROC) curves and the corresponding area under the curve (AUC) values were calculated for DM alone, APACHE II alone, a combined model including both variables, and a fully adjusted model; 95% CIs for AUCs and differences in AUCs were obtained through bootstrap resampling (1000 iterations).

A mediation analysis was planned to evaluate whether systemic complications mediated the association between DM and mortality. However, standard logistic mediation modeling could not be performed due to perfect separation, indicating near-deterministic prediction. As recommended, robust inference was derived from the observed effect structure.

All statistical analyses were conducted using SPSS version 26.0 (IBM Corp., Armonk, NY, USA) and Python 3.14 (Statsmodels and Scikit-learn libraries). A two-sided *p* value < 0.05 was considered statistically significant.

## 3. Results

Of the total 492 patients (mean age 58.6 ± 17.2 years; 50.2% female) included in this study, 98 (19.9%) had DM. Diabetic patients were significantly older (64.3 ± 14.0 vs. 57.4 ± 17.7 years, *p* = 0.0006) and more frequently had hypertension and coronary artery disease compared with non-diabetic individuals (both *p* < 0.001). The distribution of etiologies was similar between groups, whereas APACHE II scores and inflammatory markers tended to be higher in patients with diabetes (Table 1). These findings demonstrate that patients with diabetes mellitus have a higher clinical risk profile and indicate that this should be taken into consideration during the early phase of acute pancreatitis in clinical practice.

**Figure 1:**
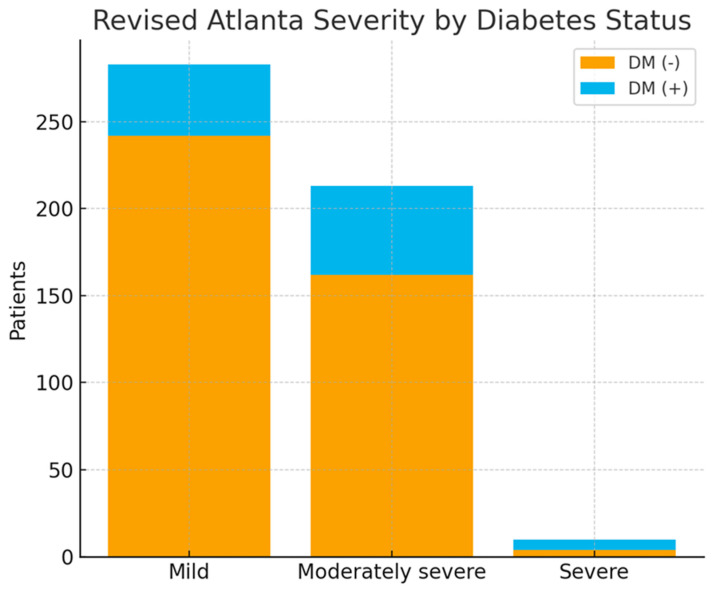
Distribution of Severity According to the Revised Atlanta Classification in Patients With and Without Diabetes. The prevalence of moderate and severe acute pancreatitis was significantly higher in patients with diabetes (*p* < 0.001). Bars represent proportions of mild, moderate, and severe disease in each group.

**Figure 2:**
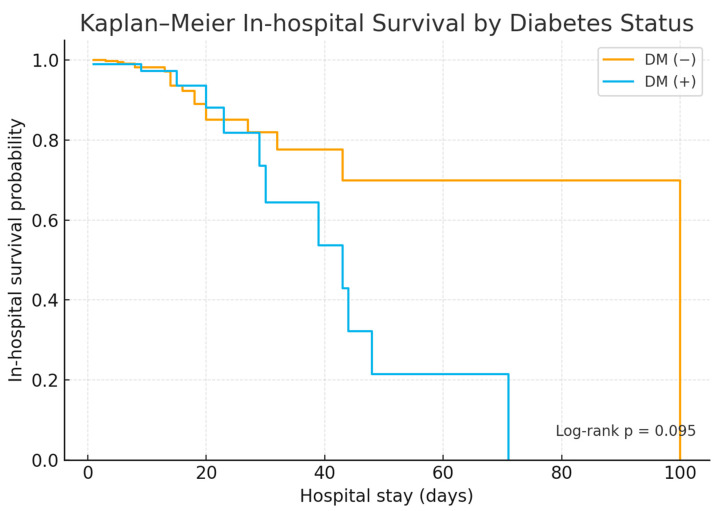
Kaplan–Meier Estimates of In-Hospital Survival According to Diabetes Status. Survival probability was lower in patients with diabetes compared with those without diabetes (log-rank *p* = 0.095), indicating a trend toward increased mortality.

Moderate-to-severe AP occurred in 67.3% of diabetic versus 37.8% of non-diabetic patients (*p* < 0.0001), and severe disease developed more frequently in the diabetic group (6.1% vs. 1.0%, *p* = 0.0057) (Table 1, Figure 1). Systemic complications were significantly more common in patients with diabetes (45.9% vs. 26.9%, *p* = 0.0004), including higher rates of renal dysfunction, infectious complications, and multiple organ dysfunction syndrome (Table 2, Table 3 and Table 4). In contrast, the occurrence of local complications did not differ significantly between the groups. Hospital mortality was higher among patients with diabetes (9.2% vs. 4.6%, *p* = 0.0344), and Kaplan–Meier analysis demonstrated numerically lower overall survival in patients with diabetes (log-rank *p* = 0.095), with early divergence in survival curves (Figure 2). Cox proportional hazards analysis confirmed diabetes as an independent predictor of in-hospital mortality (adjusted HR 2.64, 95% CI 1.17–5.97; *p* = 0.019), supporting a sustained excess risk of death throughout hospitalization.

**Figure 3:**
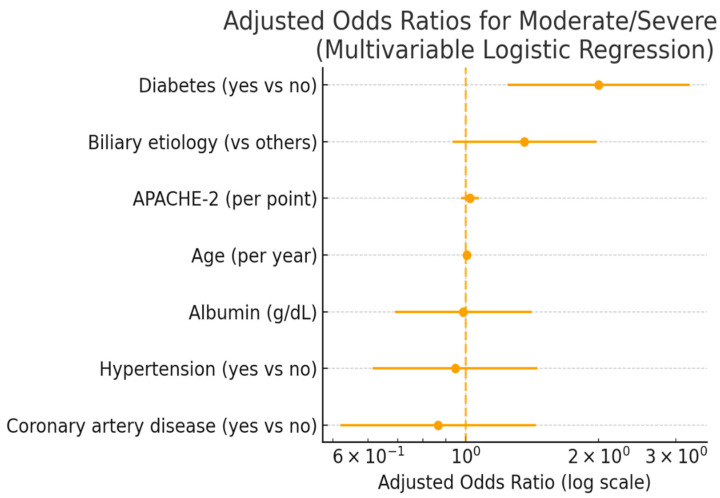
Adjusted Odds Ratios for Predictors of Moderate-to-Severe Acute Pancreatitis. Forest plot showing multivariable logistic regression results. Diabetes mellitus remained independently associated with increased risk of moderate-to-severe disease after adjusting for age, sex, hypertension, coronary artery disease, biliary etiology, and APACHE-II score. Error bars indicate 95% confidence intervals.

After adjustment for confounders, diabetes remained independently associated with the development of moderate/severe pancreatitis (adjusted OR 2.00, 95% CI 1.24–3.22; *p* = 0.004) (Table 5, Figure 3). Diabetes also independently predicted in-hospital mortality (adjusted OR 3.36, 95% CI 1.35–8.34; *p* = 0.009), along with APACHE II score (Table 6). An approximately twofold increased risk of moderate/severe pancreatitis (OR ≈ 2) and more than a threefold higher risk of mortality (OR ≈ 3.4) indicate that diabetes is an independent risk factor that markedly worsens the clinical course of acute pancreatitis.

In subgroup analyses stratified by age (<60 vs. ≥60 years), sex, etiology, and APACHE II score, the association between diabetes and both moderate/severe pancreatitis and mortality remained consistent across all subgroups (all p_interaction > 0.05), indicating that the detrimental impact of diabetes was not restricted to specific patient categories. These findings indicate that the adverse prognostic effect of diabetes is independent of age, sex, etiology, and baseline disease severity, underscoring the critical importance of early and proactive clinical management in all patients with acute pancreatitis and diabetes.

**Figure 4:**
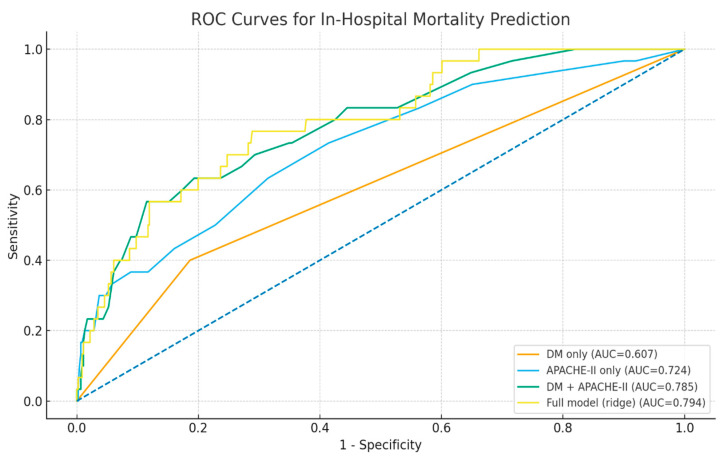
Receiver operating characteristic (ROC) curves for mortality prediction models in acute pancreatitis. ROC curves demonstrate the predictive performance of diabetes alone, APACHE-II score alone, diabetes plus APACHE-II, and the fully adjusted model, including age, sex, hypertension, coronary artery disease, and biliary etiology. The combination of diabetes and APACHE-II provided the optimal balance of parsimony and accuracy (AUC = 0.785, 95% CI 0.690–0.859), outperforming APACHE-II alone (AUC = 0.724, 95% CI 0.628–0.823). The fully adjusted model showed only marginal incremental improvement (AUC = 0.794, 95% CI 0.710–0.870). The diagonal line represents reference performance (AUC = 0.50).

In-hospital mortality prediction significantly improved when diabetes was added to APACHE II (AUC: 0.724, 95% CI 0.628–0.823 vs. AUC: 0.785, 95% CI 0.690–0.859; ΔAUC: +0.061, 95% CI −0.003 to +0.128), while the fully adjusted model provided only marginal additional improvement (AUC: 0.794, 95% CI 0.710–0.870; ΔAUC: +0.009, 95% CI −0.013 to +0.031) (Figure 4). These results show that diabetes adds prognostic value to established severity scoring without requiring complex clinical information.

A mediation analysis was planned to examine whether systemic complications mediated the association between diabetes and mortality. However, this analysis could not be performed using standard logistic modeling due to perfect separation, indicating an almost deterministic relationship between diabetes and systemic complications in AP. This strongly supports systemic complications as the primary mechanistic link connecting diabetes to increased mortality.

## 4. Discussion

In this single-center cohort of 492 patients, DM emerged as a strong determinant of disease severity and short-term mortality in AP. Patients with pre-existing diabetes were more likely to develop moderate-to-severe pancreatitis and demonstrated a higher incidence of systemic complications and in-hospital death compared with non-diabetic individuals. These associations remained independent in multivariable analysis and were consistent across prespecified clinical subgroups (age, sex, etiology, and APACHE II score), highlighting the robustness of the detrimental impact of diabetes on acute disease trajectory. Furthermore, incorporating diabetes into the APACHE II score significantly improved mortality prediction, emphasizing its role as a readily available prognostic variable.

Previous studies have reported differing results regarding the effect of DM on AP. Some studies have shown that diabetes increases the severity of AP and the risk of mortality [17,18,19], while others have suggested that this relationship is not significant [20,21]. In contrast, our comprehensive multivariate and prognostic analyses show that diabetes itself poses an excessive risk beyond traditional risk factors. Similarly to our findings, Pahomeanu and colleagues have strongly demonstrated that Type 2 DM is closely associated with AP severity, the development of systemic complications, and the need for intensive care [22]. These findings support the idea that metabolic vulnerability leads to impaired physiological resistance when exposed to acute inflammatory stress during AP [8,23].

The biological mechanisms underlying the adverse effects of diabetes on the clinical course of acute pancreatitis are multifactorial. Endothelial dysfunction, impaired micro-circulation, immune dysregulation, and an increased susceptibility to multiple organ failure represent key contributing pathways [24,25]. In the present study, the observation that the mortality burden among patients with diabetes was driven predominantly by systemic rather than local pancreatic complications suggests that diabetes amplifies the host response, leading to an exaggerated systemic inflammatory reaction and subsequent multi-organ damage. Consistent with our findings, a large meta-analysis conducted by Mikó et al. demonstrated that diabetes did not exert a statistically significant effect on local complications in acute pancreatitis (OR 1.267; 95% CI 0.964–1.659; *p* = 0.090), while it was associated with a significant increase in systemic complications and mortality [11]. Given the pronounced association between diabetes and systemic complications, standard mediation modeling could not be applied in our analysis, indicating an almost deterministic relationship and further supporting this proposed mechanistic pathway. Indeed, a previous study by Wu and colleagues also showed that early mortality in acute pancreatitis is closely linked to inflammatory and metabolic stress [26]. Taken together, these findings highlight the importance of considering diabetes-related inflammatory and metabolic dysfunctions as central mechanistic drivers of systemic decompensation, which is crucial for the interpretation of clinical outcomes in acute pancreatitis.

In our Cox regression analysis, DM was shown to independently and significantly increase the risk of in-hospital mortality by 2.5-fold, supporting a consistently high risk of death throughout the hospital stay rather than a transient early-stage effect (HR: 2.64; 95% CI: 1.17–5.97) [19].

Prognostic modeling results also showed that including diabetes in standard severity tools provided significantly increased prediction accuracy. The combined model of diabetes and APACHE II yielded better results than APACHE II alone, while additional adjustments for age and comorbidities provided only marginal improvement. Due to its simplicity and immediate clinical applicability, diabetes status can be efficiently integrated into early severity assessment algorithms to improve bedside clinical decision-making. A review of the literature reveals that no such prognostic modeling exists, although comparisons of similar models are available, albeit limited [27,28].

From a clinical perspective, these findings have important implications for the early management of acute pancreatitis in patients with diabetes. Given the consistently higher risk of systemic complications and mortality observed in diabetic patients, diabetes status should be considered an early warning marker rather than a background comorbidity. Diabetic patients with acute pancreatitis may benefit from closer hemodynamic and metabolic monitoring, a lower threshold for intensive care unit evaluation, and more aggressive early supportive management, even in the absence of overt local pancreatic complications. Furthermore, integrating diabetes status into established severity assessment tools, such as APACHE II, may enhance early risk stratification and guide timely clinical decision-making. This risk-adapted approach could help identify vulnerable patients earlier in the disease course and potentially mitigate progression to systemic decompensation.

This study has several strengths, including a relatively large and well-characterized patient population, standardized classification of disease severity using the Revised Atlanta criteria, and the application of multiple complementary analytical approaches, including logistic regression, survival analysis, subgroup stratification, and ROC-based prognostic modeling. Nevertheless, several limitations should be acknowledged. The retrospective single-center design may limit the generalizability of the findings. We were unable to classify diabetes type, lacked data on the duration of diabetes, and could not assess the role of glycemic control (e.g., HbA1c), all of which may influence risk estimates. Although the models were adjusted for major confounders, residual confounding related to cardiometabolic health cannot be fully excluded. In addition, the relatively low number of deaths may have affected the precision of effect estimates in survival analyses.

This retrospective observational study suggests that diabetes is associated with poorer clinical outcomes in acute pancreatitis; however, prospective and randomized studies are needed to establish a causal relationship.

## 5. Conclusions

In conclusion, DM is associated with substantially increased severity and in-hospital mortality in AP, primarily through an elevated risk of systemic organ failure. Incorporation of diabetes status into early severity stratification may improve prognostic accuracy and guide closer monitoring and timely interventions in this high-risk population. Prospective multicenter studies should further explore the influence of glycemic control and targeted management strategies to improve outcomes among diabetic patients with AP.

## Figures and Tables

**Table 1:** Baseline Characteristics and Clinical Outcomes of Patients with Acute Pancreatitis According to Diabetes Status.

Variable	DM (+) *n* = 98	DM (–) *n* = 394	*p* Value	OR (95% CI)
Demographics				
Age (years), mean ± SD	64.3 ± 13.3	57.4 ± 18.4	**0.0006**	—
Male gender, *n* (%)	55 (56.1)	189 (48.0)	0.1755	1.38 (0.88–2.18)
Etiology, *n* (%)				
Biliary	55 (56.1)	247 (62.7)	—	—
Post-ERCP	25 (25.5)	71 (18.0)	—	—
Idiopathic	8 (8.2)	41 (10.4)	—	—
Alcoholic	7 (7.1)	27 (6.9)	—	—
Hypertriglyceridemia	3 (3.1)	7 (1.8)	—	—
Revised Atlanta Severity Status, *n* (%)				
Mild	32 (32.7)	245 (62.2)	**<0.0001**	0.30 (0.19–0.48)
Moderate	60 (61.2)	145 (36.8)	**<0.0001**	2.79 (1.78–4.38)
Severe	6 (6.1)	4 (1.0)	**0.0057**	6.36 (1.76–22.99)
Comorbidities, *n* (%)				
Hypertension	65 (66.3)	140 (35.5)	**<0.0001**	3.58 (2.24–5.72)
Coronary artery disease	35 (35.7)	53 (13.5)	**<0.0001**	3.62 (2.17–6.02)
Pancreatitis severity				
Necrotizing pancreatitis, *n* (%)	2 (2.0)	14 (3.6)	0.7419	0.57 (0.13–2.54)
APACHE-2 score, mean ± SD	5.60 ± 4.41	5.76 ± 4.32	0.7701	—
Ranson score, mean ± SD	2.00 ± 1.74	1.92 ± 1.44	0.9935	—
BISAP score, mean ± SD	1.00 ± 1.17	0.99 ± 1.06	0.6883	—
HAPS score, mean ± SD	0.66 ± 0.62	0.63 ± 0.62	0.5896	—
Laboratory parameters				
WBC (×10^3^/μL), mean ± SD	11.5 ± 6.4	10.7 ± 4.6	0.6290	—
Hemoglobin (g/dL), mean ± SD	13.4 ± 4.0	13.2 ± 2.2	0.6431	—
Platelet (×10^3^/μL), mean ± SD	270.9 ± 82.7	265.6 ± 96.5	0.4270	—
Creatinine (mg/dL), mean ± SD	0.9 ± 0.6	1.0 ± 1.4	0.6669	—
Albumin (g/dL), mean ± SD	3.9 ± 0.6	4.0 ± 0.6	0.6542	—
CRP (mg/L), mean ± SD	44.3 ± 63.0	53.2 ± 68.4	0.3104	—
Amylase (U/L), mean ± SD	1087.6 ± 1066.9	1174.6 ± 1062.3	0.2416	—
Lipase (U/L), mean ± SD	1635.9 ± 1635.9	1820.4 ± 1734.7	0.2753	—
Clinical outcomes				
Mortality, *n* (%)	5 (9.2)	18 (4.6)	**0.0344**	2.13 (0.40–5.14)
Hospital stay (days), mean ± SD	13.4 ± 11.9	10.9 ± 10.4	**0.0042**	—

Data are presented as *n* (%) for categorical variables or mean ± SD for continuous variables. DM, diabetes mellitus; ERCP, endoscopic retrograde cholangiopancreatography; APACHE, Acute Physiology and Chronic Health Evaluation; BISAP, Bedside Index for Severity in Acute Pancreatitis; HAPS, Harmless Acute Pancreatitis Score; WBC, white blood cell; CRP, C-reactive protein; OR, odds ratio; CI, confidence interval. *p* values < 0.05 are shown in bold.

**Table 2:** Complications in Patients with Acute Pancreatitis According to Diabetes Status: General Classification.

Variable	DM (+) *n* = 98	DM (–) *n* = 394	*p* Value	OR (95% CI)
Any complication, *n* (%)	66 (67.3)	233 (59.1)	0.1464	1.43 (0.89–2.29)
Local complications, *n* (%)	30 (30.6)	104 (26.4)	0.4137	1.24 (0.76–2.01)
Systemic complications, *n* (%)	45 (45.9)	106 (26.9)	**0.0004**	2.31 (1.46–3.64)
Local complication types, *n* (%)				
Acute peripancreatic fluid collection	17 (17.3)	59 (15.0)	0.5727	1.19 (0.66–2.15)
Pseudocyst	8 (8.2)	27 (6.9)	0.6423	1.20 (0.53–2.74)
Walled-off necrosis	3 (3.1)	6 (1.5)	0.3959	2.05 (0.50–8.37)
Acute necrotic collection	1 (1.0)	7 (1.8)	1.0000	0.57 (0.07–4.72)
Pancreatic abscess	1 (1.0)	3 (0.8)	0.6041	1.34 (0.14–13.02)
Complication patterns, *n* (%)				
Local complications only	21 (21.4)	127 (32.2)	**0.0422**	0.57 (0.33–0.97)
Systemic complications only	36 (36.7)	129 (32.7)	0.4597	1.19 (0.75–1.90)
Both local and systemic	9 (9.2)	21 (5.3)	0.1692	1.80 (0.80–4.04)

Data are presented as *n* (%). Complications were classified according to the Revised Atlanta Classification. DM, diabetes mellitus; OR, odds ratio; CI, confidence interval. *p* values < 0.05 are shown in bold.

**Table 3:** Systemic Complications by Organ System Involvement in Patients with Acute Pancreatitis According to Diabetes Status.

Variable	DM (+) *n* = 98	DM (–) *n* = 394	*p* Value	OR (95% CI)
Any systemic complication	45 (45.9)	106 (26.9)	**0.0004**	2.31 (1.46–3.64)
Organ system involvement, *n* (%)				
Renal	11 (11.2)	19 (4.8)	**0.0304**	2.50 (1.15–5.43)
Pulmonary	10 (10.2)	34 (8.6)	0.6920	1.20 (0.57–2.53)
Cardiovascular	2 (2.0)	1 (0.3)	0.1026	8.19 (0.73–91.24)
Gastrointestinal	11 (11.2)	25 (6.3)	0.1262	1.87 (0.88–3.94)
Infectious	23 (23.5)	52 (13.2)	**0.0176**	2.02 (1.16–3.50)
Hematologic	3 (3.1)	7 (1.8)	0.4251	1.75 (0.44–6.88)
Neurologic	0 (0.0)	2 (0.5)	1.0000	—
Hepatic	0 (0.0)	3 (0.8)	1.0000	—
Multiple organ dysfunction syndrome	6 (6.1)	4 (1.0)	**0.0057**	6.36 (1.76–22.99)
Multiple organ involvement pattern				
No systemic complication	53 (54.1)	288 (73.1)	**0.0004**	0.43 (0.27–0.68)
Single organ system	24 (24.5)	66 (16.8)	0.0811	1.61 (0.95–2.74)
Two organ systems	9 (9.2)	26 (6.6)	0.3813	1.43 (0.65–3.16)
Three organ systems	6 (6.1)	7 (1.8)	**0.0277**	3.61 (1.18–10.98)
Four or more organ systems	0 (0.0)	1 (0.3)	1.0000	—

Data are presented as *n* (%). Systemic complications were categorized according to organ system involvement based on the Revised Atlanta Classification. DM, diabetes mellitus; OR, odds ratio; CI, confidence interval. *p* values < 0.05 are shown in bold.

**Table 4:** Specific Types of Systemic Complications in Patients with Acute Pancreatitis According to Diabetes Status.

Complication Type	DM (+) *n* = 98	DM (–) *n* = 394	*p* Value	OR (95% CI)
Sepsis	21 (21.4)	48 (12.2)	**0.0229**	1.97 (1.11–3.47)
Acute kidney injury	11 (11.2)	17 (4.3)	**0.0136**	2.80 (1.27–6.20)
Pleural effusion	7 (7.1)	26 (6.6)	0.8229	1.09 (0.46–2.59)
Ascites	11 (11.2)	22 (5.6)	0.0675	2.14 (1.00–4.57)
Urinary tract infection	3 (3.1)	4 (1.0)	0.1452	3.08 (0.68–13.99)
Thrombosis	3 (3.1)	7 (1.8)	0.4251	1.75 (0.44–6.88)
Respiratory failure	4 (4.1)	5 (1.3)	0.0827	3.31 (0.87–12.57)
Acute kidney disease	0 (0.0)	2 (0.5)	1.0000	—
Toxic hepatitis	0 (0.0)	3 (0.8)	1.0000	—

Data are presented as *n* (%). DM, diabetes mellitus; OR, odds ratio; CI, confidence interval. *p* values < 0.05 are shown in bold.

**Table 5:** Factors Associated with Moderate-to-Severe Acute Pancreatitis in Multivariable Logistic Regression Analysis.

	Adjusted OR	95% CI	*p* Value
Diabetes (yes vs. no)	2.0	1.24–3.22	0.004
Age (per year)	1.01	0.99–1.02	0.338
Hypertension (yes vs. no)	0.95	0.62–1.46	0.801
Coronary artery disease (yes vs. no)	0.86	0.52–1.44	0.579
Biliary etiology (vs. others)	1.36	0.93–1.98	0.11
APACHE-II (per point)	1.02	0.97–1.07	0.377
Albumin (g/dL)	0.99	0.69–1.41	0.944

OR = odds ratio; CI = confidence interval; aOR values adjusted for age, sex, hypertension, coronary artery disease, biliary etiology, and APACHE-II score. Moderate/severe acute pancreatitis is defined according to the Revised Atlanta classification.

**Table 6:** Independent Predictors of In-Hospital Mortality in Patients With Acute Pancreatitis: Multivariable Logistic Regression Analysis.

	Adjusted OR	95% CI	*p* Value
Diabetes (yes vs. no)	3.36	1.35–8.34	0.009
Age (per year)	1.01	0.98–1.04	0.57
Hypertension (yes vs. no)	0.73	0.27–1.98	0.535
Coronary artery disease (yes vs. no)	1.37	0.47–3.99	0.562
Biliary etiology (vs. others)	1.11	0.46–2.65	0.822
APACHE-II (per point)	1.14	1.05–1.23	0.001
Albumin (g/dL)	0.3	0.16–0.60	0.001

OR = odds ratio; CI = confidence interval; aOR values adjusted for age, sex, hypertension, coronary artery disease, biliary etiology, and APACHE-II score. Mortality is defined as in-hospital death.

## Data Availability

The data underlying this article will be shared upon reasonable request to the corresponding author.
